# Control and eradication of porcine reproductive and respiratory syndrome virus type 2 using a modified-live type 2 vaccine in combination with a load, close, homogenise model: an area elimination study

**DOI:** 10.1186/s13028-016-0270-z

**Published:** 2017-01-05

**Authors:** Poul H. Rathkjen, Johannes Dall

**Affiliations:** 1 Boehringer Ingelheim Vetmedica GmbH, Binger Straße 173, 55216 Ingelheim, Germany; 2 PORCUS svinefagdyrlaeger og agronomer, Oerbaekvej 276, 5220 Odense, Denmark

**Keywords:** Area regional control, Elimination, Modified-live vaccine, Load close homogenise, PRRS

## Abstract

**Background:**

Porcine reproductive and respiratory syndrome virus (PRRSV) causes significant animal and economic losses worldwide. The infection is difficult to control and PRRSV elimination at local level requires coordinated intervention among multiple farms. This case study describes a successful elimination of PRRSV from all 12 herds on the Horne Peninsula, Denmark, using a combination of load, close, homogenise (LCH) using PRRSV type 2 modified-live vaccine, optimised pig flow, and’10 Golden Rules’ (10GR) for biosecurity management. To the authors’ knowledge, this is the first successful European PRRSV area elimination project documented in detail. The PRRSV type 2 modified-live vaccine was used as part of the LCH method in breeding herds. Complete or partial depopulation was performed in some infected herds. A simplified biosecurity protocol (10GR) based on the McREBEL™ system of pig flow management, was employed in all herds and at all times throughout the study.

**Results:**

At study commencement, all herds were infected with PRRSV, and most were actively shedding virus. In just over 18 months, all 12 herds on the Horne Peninsula were confirmed to be PRRSV negative by polymerase chain reaction testing and negative for antibodies against PRRSV by enzyme–linked immunosorbent assay testing. All herds were subsequently obtained ‘Specific Pathogen Free’ status for PRRSV.

**Conclusions:**

This study provides compelling evidence suggesting that an area elimination plan combining LCH with PRRSV type 2 vaccination, optimised pig flow, and 10GR for biosecurity management can effectively eliminate PRRSV from a geographic area. Additionally this study confirms the value of a previously unpublished, simplified alternative to the McREBEL system for controlling PRRSV.

**Electronic supplementary material:**

The online version of this article (doi:10.1186/s13028-016-0270-z) contains supplementary material, which is available to authorized users.

## Background

Porcine reproductive and respiratory syndrome (PRRS) is one of the most prevalent viral swine diseases in the world, responsible for substantial economic losses worldwide [1]. In the US, PRRS is estimated to cause annual losses of around $664 million [2]. A 2012 economic analysis in nine Dutch sow herds found that the mean economic loss per sow per 18-week outbreak of PRRSV was €126 [3].

Porcine reproductive and respiratory syndrome is caused by the PRRS virus (PRRSV) and was first reported in the late 1980s [4]. Two PRRSV genotypes have been described: type 1 and type 2, isolated in Europe and North America, respectively. Sequence comparison has highlighted significant genetic differences between them [5].

Porcine reproductive and respiratory syndrome causes high morbidity and mortality, poor reproductive performance and slow piglet growth rates [1]. The extent of reproductive symptoms varies depending on age, pregnancy status and stage of gestation [6, 7]. In non-pregnant sows, PRRS can develop without symptoms, or cause appetite loss or fever [6]. In pregnant sows, the virus may cross the placenta during late gestation, infect developing foetuses and increase the risk of abortion, early farrowing and foetal death [8, 9]. Neonatal and nursery pigs may experience respiratory distress, listlessness, pneumonia, high fever, anorexia, conjunctivitis and growth retardation [6, 10–12]. In growing and finishing pigs, the severity of PRRS varies from no detectable signs to fatal pneumonia, depending on the viral strain and the presence of opportunistic bacterial or viral coinfections [13].

At both herd and individual level, PRRSV infection is difficult to control for several reasons. PRRSV infection can be completely cleared by the porcine immune system, but considerable gaps remain in our understanding of the immunological response to PRRSV [14]. In the field, the diversity of PRRSV is increasing [15]. Levels of genetic similarity between vaccine and field challenge have often been used as a predictor of vaccine efficacy, but the ability of a vaccine to protect against a certain field virus is not linked to the level of sequence homology it shares with the challenging strain: the degree of genetic similarity does not predict the cross-protective ability of the vaccine [14]. Despite these challenges, vaccination is a popular method of controlling PRRS and reducing losses caused by it. Multiple vaccines are commercially available [11].

Accepted PRRSV control and elimination models for multiple herds include herd closure with either total herd replacement or with normal herd replacement rates, and depopulation/repopulation of infected herds [16, 17]. Herd closure involves preventing entry of new animals, while depopulation/repopulation involves complete removal of PRRSV-positive animals from a herd, cleaning and decontaminating the site, then replacing with PRRSV–negative animals bred elsewhere [17]. Depopulation/repopulation is effective, but expensive because of requirements for large external breeding projects [17] and loss of productivity after depopulation [2]. Alternatively, the load, close, homogenise (LCH) (also known as load, close, expose) model allows the PRRSV status to stabilise in a breeding herd before introduction of new PRRSV-negative animals [18, 19]. Using this model, PRRSV can be completely eliminated from large breeding (sow) herds [14] without incurring substantial losses of productive time (i.e. time without weaned pigs) for the breeding herd. LCH is accomplished by loading herds with gilts before closing the herds to new animals for minimum of 200 days [14]. Uniform PRRS status must then be achieved either by simultaneous vaccination or by inoculation with serum containing resident virus [19]. The LCH model is inexpensive compared with depopulation/repopulation of a breeding stock [20], and broadly recognised as effective at stabilising PRRSV-positive breeding herds [21, 22], but it requires stringent biosecurity measures to prevent virus transmission within the herd.

The Management Changes to Reduce Exposure to Bacteria to Eliminate Losses™ (McREBEL) system was developed in 1994 to reduce the spread of PRRSV and secondary bacterial infections among farrowing house pigs, and to nursery pigs [22–24]. The McREBEL system helps stabilise PRRSV infection within the breeding herd and reduce mortality among infected nursery pigs [24]. The McREBEL system has several advantages, but the system can be difficult to implement for many reasons. For example, farm staff can be unwilling to abandon cross-fostering and perform piglet euthanasia, and staff incentive plans need to be reviewed to ensure its success [24].

The Horne Peninsula is a region in the southern Danish island of Funen, approximately 50 kilometres southwest of Odense. The peninsula is a naturally limited geographical area; it is surrounded with water on three sides, and spans approximately 6 kilometres North to South, and 10 kilometres East to West. It is an area with intensive pig farming: 12 herds are situated on the peninsula, and include breeding, wean-to-finish and finishing production, but no other herds are situated within 4 km.

Until the current elimination plan started, all farms on the Horne Peninsula repeatedly experienced PRRS outbreaks despite multiple attempts to control the virus. Common problems were periodic outbreaks of abortion, many stillborn piglets, poorly-lactating sows, poorly–performing piglets at weaning, and high mortality in one particular finisher herd. Different attempts to control the PRRSV in the area had already been tried, but with low success. Prior control attempts included depopulation, vaccinating incoming gilts with PRRS modified-live vaccine (MLV) in quarantine and systematically implementing some McREBEL rules to varying degrees. Overall, a systematic approach to control or eliminate PRRSV from the whole area was needed.

The objective of this area elimination case study was to eliminate PRRSV infection as defined by absence of pigs with PRRSV and corresponding antibodies from all herds on the Horne Peninsula, Denmark, using a combination of LCH using PRRS modified-live type 2 vaccine, optimised pig flow, and implementation of’10 Golden Rules’ (10GR) for biosecurity management.

## Methods

### Herds

The study area included all 12 herds on the Horne Peninsula: five finisher herds, four breeding herds, two wean-to-finish herds, and one gilt quarantine (Table 1). Breeding herds contained sows, gilts ready for breeding, and weaned piglets. Wean-to-finish herds received weaned piglets from breeding herds and raised them until slaughter. Finisher herds received piglets at around 11 weeks of age, and raised them until slaughter.

**Table 1:** Overview of herds included in the study

Herd name	Owner	Type of production	Number and type of animals	Age ranges, weeks	Approximate weight ranges, kg
Flow 1					
F1B1	Owner 1	Breeding	500 sows	Piglets: 0–4	Piglets: 1–7
F1B2	Owner 1	Breeding	300 sows	Piglets: 0–4 Weaned piglets: 4–12	1–7, 5–30
F1Q	Owner 1	Gilt quarantine	200 pregnant sows 550 gilts 1000 finishers	10–32 12–18	30–120 30–110
F1WF1	Owner 2	Wean-to-finish	1220 finishers	4–18	7–110
F1WF2	Owner 3	Wean-to-finish	2000 finishers	4–18	7–110
F1F1	Owner 2	Finishing	1000 finishers	11–18	30–110
F1F2	Owner 4	Finishing	800 finishers	11–18	30–110
Flow 2					
F2B1	Owner 5	Breeding	400 sows 2000 growers	Piglets: 1–4 Weaned piglets: 4–12	1–7 5–30
F2B2	Owner 5	Breeding	320 sows 1300 growers	Piglets 1–4 Weaned piglets: 4–12	1–7 5–30
F2F1	Owner 6	Finishing	1600 finishers	11–18	30–110
F2F2	Owner 7	Finishing	900 finishers	11–18	30–110
F2F3	Owner 5	Finishing	1000 finishers	11–18	30–110

*F1B1* Flow 1 Breeding Herd 1, *F1B2* Flow 1 Breeding Herd 2, *F1F1* Flow 1 Finisher Herd 1, *F1F2* Flow 1 Finisher Herd 2, *F1Q* Flow 1 Quarantine, *F1WF1* Flow 1 Wean-Finish 1, *F1WF2* Flow 1 Wean-Finish 2, *F2B1* Flow 2 Breeding Herd 1, *F2B2* Flow 2 Breeding Herd 2, *F2F1* Flow 2 Finisher Herd 1, *F2F2* Flow 2 Finisher Herd 2, *F2F3* Flow 2 Finisher Herd 3

In total, the herds on the peninsula contained approximately 15,000 animals. Movement of animals between the 12 herds was coordinated in two separate pig flows: Flow 1 and Flow 2. All animals in Flow 1 originated from F1B1 and F1B2, and all animals in Flow 2 originated from F2B1 and F2B2. The herds in each flow were controlled by four separate owners who worked closely with each other. PRRSV-negative gilts were imported into F2B1 only after completing 12 weeks in all in, all out (AIAO) quarantine: no other herds received animals from outside of the Horne Peninsula. Animals were exported out of the Horne Peninsula from the nursery of F1B2 only. All other animal movements were within the herds on the peninsula.

### Layout of farm buildings

Breeding herds contained separate areas: farrowing rooms and nursery rooms. F1WF1 had four nursery rooms and six finisher rooms: all were separate, but all pigs entered through one nursery room and passed through others whilst in transit. Similarly, pigs moving from nursery to finisher rooms passed through several rooms containing piglets of other ages. At study commencement, F1WF1 operated as continuous flow. F1WF2 comprised two barns: a nursery and a finishing barn, both of which had multiple rooms. AIAO production was observed in all rooms in both the nursery and finishing barns. Finishing herds contained pigs separated into different rooms by age group, and AIAO production was observed. F1Q consisted of two adjacent buildings, connected by corridors. One building housed pregnant sows and finishers that arrived from F1B2, and the other building housed gilts in acclimatisation and quarantine. Separate rooms were entered from the corridor, and rooms did not share airspace and were not connected under the floor slats. Strict AIAO production was observed.

### Study timeframe

The study began in the first week of July, 2013.

### Week 0

Load close homogenise was commenced at Week 0 in F1B1 and F1B2. At Week 0, F1Q was loaded with gilts 10–32 weeks of age, and sites with sows and gilts were closed for the next 29 weeks. All sows, gilts (existing and newly-introduced), boars and piglets (older than 1 week) on all sites except F2B1 and F2B2 were homogenised by vaccination with 2 ml PRRSV type 2 MLV (Ingelvac® Boehringer Ingelheim Vetmedica Inc., St. Joseph, MO, USA). F2B1 and F2B2 were already PRRSV positive-stable at study commencement, so homogenisation was deemed unnecessary. From Weeks 0–10, Finisher pigs in F2F1, F2F3 and F2F3 were vaccinated with 2 ml PRRS type 2 MLV upon arrival from F2B1 and F2B2, to avoid introducing naïve pigs. Vaccinations were performed according to the manufacturer’s guidelines on dose and administration (Boehringer Ingelheim Vetmedica GmbH, Germany).

Depopulation commenced in F2B1 and F2B2. The nursery rooms containing the two oldest age groups (piglets older than 8 weeks) were depopulated.

### Weeks 2–4

All piglets in F1B1 and F1B2 were vaccinated with 2 ml PRRSV type 2 MLV when they reached 7 days of age. Vaccination of sows, boars and gilts was repeated at Week 4. All animals in F2F1, F2F2 and F2F3 that had not been vaccinated previously were also vaccinated at Week 4.

### Weeks 6–16

On a rolling basis from Week 6 to 16, all weaned piglets (3 weeks of age) that had not already been vaccinated when entering breeding herd nurseries or wean-to-finish nurseries, were vaccinated with 2 ml PRRSV type 2 MLV upon arrival.

At Week 16, depopulation of nursery rooms in F1B2, and partial depopulation of nursery rooms in F1WF1 commenced.

### All times throughout the study

Sampling and diagnostic testing to determine PRRSV shedding and exposure status continued every 5 weeks from study commencement, until all herds were confirmed PRRSV and antibody negative by polymerase chain reaction (PCR) and enzyme-linked immunosorbent assay (ELISA), respectively.

The 10GR for biosecurity and pig flow management were employed in all herds and at all times throughout the study (Table 2). These rules were devised in 2005 by Boehringer Ingelheim, and are based on the principles of the McREBEL system for disease management [23].

**Table 2:** The 10 Golden Rules

	Rule	Rationale
1	Minimise cross-fostering and movement of piglets: cross-foster only surplus piglets	The immune system is immature in newborn piglets; immunity depends on passive immunisation transmitted via colostrum [37]. Piglets receive optimal protection from their own mothers so should only be moved if a sow cannot support her whole litter. Furthermore, moving piglets to other sows causes weight loss in both moved piglets and their new litter mates [38]
2	Avoid cross-fostering after 48 h	Maternal immune protection starts to decrease when piglets reach 3 days of age [37]. Cross-fostering before maternal protection decreases is strongly recommended
3	Avoid spreading disease when handling piglets by keeping piglets in pens	Urine, blood, faeces and semen are vehicles for PRRSV transmission; special attention should be paid to the use of equipment (e.g. needles and castration equipment)
4	Change needles between litters	PRRSV is easily transmitted among pigs by needles, so regular replacement of needles (at least between litters) is recommended. Diseased piglets should be treated after healthy piglets
5	Do not move diseased piglets	Diseased piglets often have compromised immunity and comorbidities that increase the likelihood that they are also carrying PRRSV. Their viral load is also likely to be higher, increasing the risk of spreading infection. Therefore diseased piglets should remain with the same sow to limit viral spread: if a piglet is too weak for this, it should be euthanised
6	Wean all piglets from each batch simultaneously, and ban weaned piglets from the farrowing rooms	Holding smaller piglets back in the farrowing rooms for quality before they are weaned can jeopardise PRRS control programmes [39]. Such piglets are more likely to be diseased, and to spread PRRSV to others
7	Maintain strict AIAO batch production at all times from weaning to finishing	After piglets are weaned, batch production should continue, and should be either by site, barn or room. If a batch is not completely removed before placement of new pigs, infection pressure rapidly increases. Do not share needles, equipment, personnel and protective equipment between batches (unless cleaned and disinfected)
8	Avoid contact between age groups	Risk of infection is increased 13-fold if contact is permitted between growing pigs of different ages during restocking of rooms [40]. Mixing PRRSV-positive pigs in one age group with PRRSV-negative, non-vaccinated pigs in other age groups greatly increases PRRSV shedding [41, 42]
9	Avoid contact between sows and piglets (<6 months of age)	Breeding herds and grower/finisher pigs should never be in contact (i.e. when moving pigs and sows around the farm) because cross-contamination between groups can occur
10	Introduce incoming and home-produced gilts via quarantine. Administer PRRSV MLV upon entry to quarantine areas	Natural immunisation of gilts should be avoided because it cannot be monitored or controlled. If natural immunisation occurred just before entering a breeding site, there would be a high risk of introducing wild-type PRRSV to the breeding herd. While in quarantine, gilts should be immunised twice with PRRS MLV (vaccinations should be administered 4 weeks apart)

*AIAO* all in all out, *MLV* modified-live vaccine, *PRRSV* porcine reproductive and respiratory syndrome virus

## 10 Golden Rules for biosecurity management

Staff members received training in the 10GR from the responsible veterinarian on each farm. Training emphasised the importance of open and frequent communication among staff members. To ensure optimal compliance with the 10GR, farms were audited by the farm veterinarian at 5-week intervals throughout the study. If the audit found that the 10GR were not being followed, this was communicated to the staff, and corrected.

### Sampling and diagnostic testing of PRRSV status

Piglets were randomly selected from among all parity sows. To determine PRRSV status among weaning-age piglets 8 weeks before study commencement, blood samples were taken from 3-week old (pre-wean) piglets, and piglets 2, 3, 4, 6, 7 and 8 weeks after weaning in breeding herd nurseries. Samples were then taken at 5-week intervals throughout the study. Serum was harvested from the blood samples by routine methods.

In breeding and WF herds, blood samples were taken from at least 30 animals at each time point, and comprised samples from a minimum of 5 animals per age group (each week of age). These sample sizes were adequate to detect at least one positive sample with 95% confidence if the prevalence of PRRSV positive pigs was 10% or higher [25], and to meet the sample size requirements needed for declaring of PRRSV free Specific Pathogen Free (SPF) status [26].

In finisher herds, blood samples were taken from at least 20 animals. This sample size was adequate to detect at least one positive sample with 95% confidence if the prevalence of PRRSV positive pigs was 15% or higher. Fewer samples were taken from finisher herds than from breeding and WF herds because it was assumed that if pigs were infected with PRRSV during the early finishing period, the prevalence of infected pigs would be higher. This sample size also met the sample size requirements needed for declaration of PRRS free SPF status in routine monitoring of negative herds.

Individual serum samples were used to evaluate PRRSV exposure status (indicated by the presence of PRRSV antibodies in serum). An ELISA method (IDEXX HerdCheck PRRS X3 ELISA, IDEXX Laboratories Inc., Westbrook, ME, USA) was used to detect PRRSV antibodies. Serum samples from each age group were pooled, and used to determine PRRSV shedding status (indicated by the presence of viral DNA in serum). Reverse transcriptase PCR (rtPCR) was used to detect PRRSV RNA. Combining PCR and ELISA increased the confidence that detection would occur if pigs were exposed to PRRSV.

A herd was declared to have a positive exposure status (ELISA positive; presence of anti-PRRSV antibodies) if one or more individual serum samples was positive (Sample: Positive ratio cut off >0.4). A herd was declared to have a positive shedding status (PCR positive; presence of PRRSV RNA) if one or more pooled serum samples was PCR positive for PRRSV RNA. PRRSV was considered eliminated from a herd after PRRSV RNA or antibodies were not detected after testing at four consecutive time points (taken at 5 week intervals).

### PRRS status of herds, and official declaration of PRRSV Specific Pathogen Free status

Throughout the study, overall PRRS status of herds throughout the study was classified according to the American Association of Swine Veterinarians (AASV) terminology, taking into account both PRRSV shedding and exposure status [27]. Herds were classified as either: negative (ELISA negative and PCR negative), positive-stable (ELISA positive but PCR negative); or positive-unstable (ELISA positive and PCR positive).

In addition, declaration of PRRSV free SPF status was sought, according to the regulations from SPF–SUS, Denmark [26]. PRRSV SPF status can be granted only when PRRSV has been eliminated (proven PCR and ELISA negative) from a herd. To meet the requirements for PRRSV free SPF declaration, 30 PRRSV-negative sentinel gilts were placed into each herd after samples from herds tested both PCR and ELISA negative. PRRSV free SPF status was confirmed if the sentinels remained PCR and ELISA negative after 6 months.

## Results

### Time taken to eliminate PRRSV from all farms on the Horne Peninsula

**Fig. 1:**
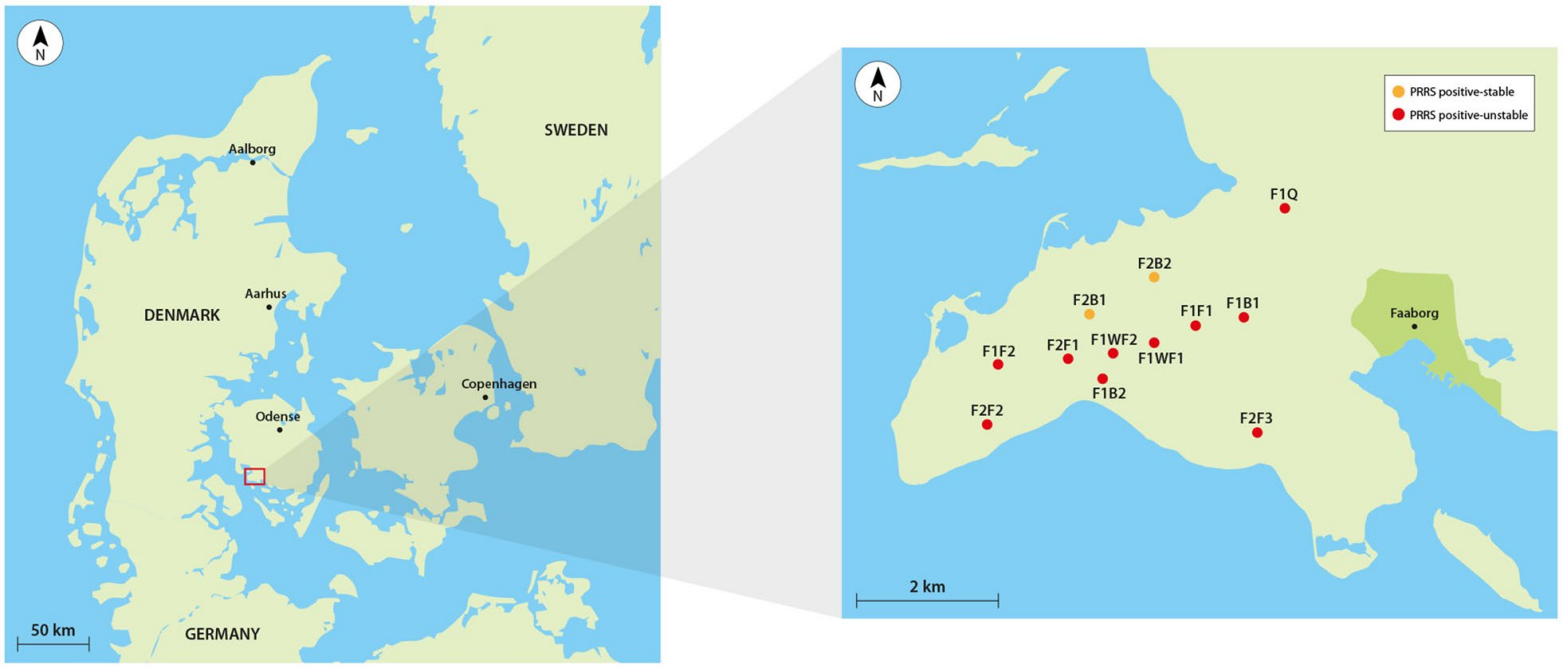
Locations of herds on the Horne Peninsula, and PRRSV status at study commencement. *F1B1* Flow 1 Breeding Herd 1, *F1B2* Flow 1 Breeding Herd 2, *F1F1* Flow 1 Finisher Herd 1, *F1F2* Flow 1 Finisher Herd 2, *F1Q* Flow 1 Quarantine, *F1WF1* Flow 1 Wean-Finish 1, *F1WF2* Flow 1 Wean-Finish 2, *F2B1* Flow 2 Breeding Herd 1, *F2B2* Flow 2 Breeding Herd 2, *F2F1* Flow 2 Finisher Herd 1, *F2F2* Flow 2 Finisher Herd 2, *F2F3* Flow 2 Finisher Herd 3, *PRRS* porcine reproductive and respiratory syndrome

The study extended from July 2013 to July 2015. All herds on the Horne Peninsula were initially PRRSV positive-unstable except F2B1 and F2B2, which were positive-stable (Fig. 1; Additional file 1). All herds had confirmed PRRSV free SPF status by April 2015; less than 2 years after study commencement (Table 3).

**Table 3:** Time for herds to obtain official PRRS free SPF status

Herd	PRRS free SPF status achieved	Notes
F1B1	January 2015	July 2013: weaned ELISA and PCR positive piglets at study commencement September 2013: weaned PCR negative but ELISA positive piglets July 2014: three-week old piglets remained ELISA positive until July 2014
F1B2	January 2015	July 2013: weaned ELISA and PCR positive piglets at study commencement September 2013: weaned PCR negative but ELISA positive piglets November 2013: sentinels were about to be introduced, but two age groups tested PCR positive (5-week old and 7-week old piglets in nursery rooms). Sequencing revealed 99.17% homology to PRRS type 2 MLV The nursery was depopulated to prevent PRRS spreading to the sows: Oldest pigs (26–32 kg) were exported out of the area Younger pigs (14–26 kg) were moved to isolation rooms in F1Q, vaccinated, then eventually slaughtered Piglets considered to be PCR negative were moved to F1WF2. (these were the only extra facilities available) The nursery was cleaned and disinfected before repopulation November 2014: two samples were close to the ELISA assay cut-off (SP > 0.4) March 2015: one sample was ELISA positive, but simultaneously PCR negative. This was assumed to be false positive
F1WF1	Finishers depopulated in February 2015	October 2013: partially depopulated October 2013–February 2014: ≤20% of samples tested from piglets were ELISA positive until age 6–7 weeks. All samples were PCR negative 100% of pigs older than 7 weeks were ELISA and PCR negative February 2014: samples from 17-week old piglets were ELISA positive, but PCR negative March 2014: 16- and 18-week pigs were found to be PCR and ELISA positive April 2014: finisher rooms partially depopulated again. The site then remained PCR and ELISA negative until October 2014 October 2014: samples from 17- to 18-week old piglets were ELISA and PCR positive (possibly from F1WF2) December 2014: samples from 11-week old piglets were ELISA negative, PCR positive. Samples from 13- to 15-week old piglets were both ELISA and PCR positive January 2015: Total depopulation
F1WF2	January 2015	November 2013: received a batch of PCR positive pigs from F1B2 (although these were considered PRRS negative when moved). Lack of compliance with Golden Rule 8 meant the finisher rooms were continuously PCR positive until October 2014 October 2014: finisher barn depopulated, but infection probably spread to nearby F1WF1. Gradual repopulation from nursery. Herd then remained PCR and ELISA negative for the remainder of the study
F1F1	April 2015	October 2013: received depopulated (30 kg) pigs from F1WF1. Samples tested ELISA and PCR positive until March 2015, until the whole herd was depopulated March 2015: repopulated
F1F2	January 2014	November 2013: received PRRS type 2 MLV vaccinated pigs from F1WF1 until October 2013. Partial depopulation. Received pigs from F1WF1 since November 2013 on an AIAO basis.
F1Q	January 2015	July 2013. Mass vaccination of all gilts and sows (two times, 4 weeks apart, according to same schedule as in F1B1 and F1B2). Gilts remained in quarantine for 12 weeks. These gilts had been transferred to breeding herds by December 2013 November 2013: received pigs 14–26 kg from F1B2. These pigs were placed in an isolated room, vaccinated with PRRS type 2 MLV, then later slaughtered to prevent PRRSV from spreading to the rest of the site January 2014: PRRS negative gilts bred elsewhere were introduced to gilt quarantine April 2014: acclimatised (external) gilts were moved to breeding herds
F2B1	July 2014	July 2013: weaned PCR negative piglets at study commencement
F2B2	July 2014	July 2013: weaned PCR negative piglets at study commencement. Nursery rooms containing oldest two age groups were depopulated
F2F1	August 2015 (but no PRRS positive pigs since October 2013)	July 2013: received PCR positive piglets from F2B2 at study commencement. From Weeks 0–10, all finisher pigs were vaccinated after introduction. Partially depopulated, and then only received PRRS negative animals October 2013: samples tested PCR negative, and remained negative for the remainder of the study
F2F2	November 2013	July 2013: received PCR positive piglets from F2B2 at study commencement From Weeks 0–10, all finisher pigs were vaccinated after introduction. Partially depopulated, then received only PRRS negative animals November 2013: samples tested PCR negative, and remained negative for the remainder of the study
F2F3	November 2013	July 2013: received PCR positive piglets from F2B2 at study commencement. From Weeks 0–10, all finisher pigs were vaccinated after introduction. Partially depopulated, then received only PRRS negative animals November 2013: samples tested PCR negative, and remained negative for the remainder of the study

Study commenced in July 2013. Positive-unstable defined as ELISA positive for PRRS antibody, and PCR positive for PRRSV RNA (actively shedding); positive-stable defined as ELISA positive for PRRS antibody in serum but PCR negative (not shedding)

*F1B1* Flow 1 Breeding Herd 1, *F1B2* Flow 1 Breeding Herd 2, *F1F1* Flow 1 Finisher Herd 1, *F1F2* Flow 1 Finisher Herd 2, *F1Q* Flow 1 Quarantine, *F1WF1* Flow 1 Wean-Finish 1, *F1WF2* Flow 1 Wean-Finish 2, *F2B1* Flow 2 Breeding Herd 1, *F2B2* Flow 2 Breeding Herd 2, *F2F1* Flow 2 Finisher Herd 1, *F2F2* Flow 2 Finisher Herd 2, *F2F3* Flow 2 Finisher Herd 3, *PRRS* porcine reproductive and respiratory syndrome

### Elimination in breeding herds

F1B1 and F1B2 were initially weaning PCR and ELISA positive piglets. By September 2013, both were weaning PCR negative, but ELISA positive piglets. Three-week old piglets remained ELISA positive on all sampling points until July 2014 (51 weeks after LCH was implemented). These antibodies were presumed to be maternal because no samples were PCR positive at the same time points.

Virus was detected in 5-week old and 7-week old piglets in the F1B2 nursery in November 2013. The virus was isolated from the ELISA and PCR positive piglets in F1B2, and the virus gene open reading frame 5 (ORF-5) was sequenced (Bioscreen GmBH, Hannover, Germany), and shown to have 99.17% sequence homology to the PRRSV type 2 MLV strain. The nursery was depopulated to prevent the virus spreading to the sows. The oldest pigs (26–32 kg) were exported out of the peninsula, but the youngest pigs (14–26 kg; too small to be sold) were moved to isolation rooms in F1Q, where they were vaccinated and slaughtered at a later time point. Remaining piglets that were considered to be negative were moved to F1WF2. The empty nursery was cleaned and disinfected before repopulation, and no virus was subsequently detected on the site.

In November 2014, two samples (both from F1B2) tested close to the ELISA assay cut-off, and in March 2015, another sample (from F1B2) tested ELISA positive. None of these samples were simultaneously PCR positive so all were assumed to be false-positives (Additional file 2).

**Fig. 2:**
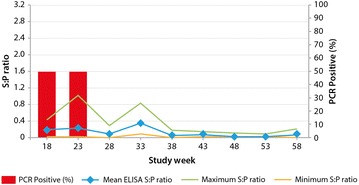
PRRSV ELISA and PCR monitoring of 10-week old piglets in F1B1 and F1B2. A minimum of 5 samples were taken at each sampling point. ELISA was performed on individual samples; PCR was performed on a pooled sample at each time point. *ELISA* enzyme-linked immunosorbent assay, *PCR* polymerase chain reaction

PCR testing of samples from 10 week old piglets in F1B1 and F1B2 nurseries revealed that PRRSV remained present until Week 23 (Fig. 2). No virus was detected in any 10-week old piglets from Week 28 onwards.

At study commencement, F2B1 and F2B2 were PRRSV positive-stable, and weaning PRRSV PCR negative piglets. Piglets became PCR positive in the later nursery rooms, so the two rooms containing the oldest age groups were depopulated. F2B1 and F2B2 received PRRSV free SPF status in July 2014.

### Elimination in wean-to-finish and finisher herds

F1WF1 was partially depopulated in October 2013, after piglet vaccination (at weaning) stopped, and then all piglets tested PCR negative until February 2014. Up to 20% of piglets continued to test ELISA positive until age 6–7 weeks, probably due to the presence of maternal antibodies (Additional file 3).

F1WF2 received a batch of presumed PCR negative piglets from F1B2 in November 2013, but PCR positive piglets were detected shortly afterwards. Despite regular auditing of procedures by the veterinarian, staff were not able to comply with Golden Rule 8 (avoid contact between age groups; Table 2). This resulted in finisher rooms remaining continuously PCR positive until they were depopulated in October 2014.

F1F2 remained PCR positive until November 2013; 5 months after study commencement, and received PRRSV free SPF status in April 2014. F2F1 tested PCR negative in October 2013, and F2F2 and F2F3 tested PCR negative one month later, and remained both PCR and ELISA negative for the remainder of the study. PRRSV free SPF status was declared in October 2013 for F2F1, and in November 2013 for F2F2 and F2F3.

### Re-infection in F1WF1 and F1F1

In October 2014, just before PRRSV free SPF status was to be declared for F1WF1, and at the same time that the finisher rooms of F1WF2 were depopulated due to reinfection, 20 and 100% of samples from 17- to 18-week old piglets, respectively, tested positive by ELISA, and pooled samples from both age groups were PCR positive (Additional file 3). Three months later, PRRSV had spread to nearby F1F1, which had also been close to PRRSV elimination. The re-infection prompted full depopulation of both sites, and no new pigs were introduced until March 2015. No further samples tested either ELISA or PCR positive after repopulation. F1F1 was the last on the peninsula to achieve PRRSV free SPF status, in April 2015.

## Discussion

The objective of the area elimination case study reported here was to eliminate PRRSV from all herds on the Horne Peninsula, Denmark, using a combination of LCH using PRRSV type 2 MLV, optimised pig flow, and implementation of the 10GR for biosecurity management. This study shows that these techniques, in combination, successfully eliminated PRRSV from all herds on the Horne Peninsula, Denmark, according to Danish SPF-SUS regulations [26]. Eighteen months later (November 2016), all herds still retain PRRSV free SPF status. To the authors’ knowledge, this is the first successful European PRRSV area elimination project documented in detail.

Throughout the study, overall PRRS status of herds was classified according to the AASV terminology, and then PRRS was deemed eliminated from a herd when PRRS free SPF status was declared, according to the regulations from SPF–SUS, Denmark [26]. The use of AASV terminology throughout the study enabled herd status to be monitored month by month, thus allowing rapid response to re-infection. PRRS free SPF status was sought to fetch the maximum price when the pigs were sold.

At study commencement, all herds in both flows tested PCR positive for PRRSV infection according to AASV terminology [27], and all except F2B1 and F2B2 were positive-unstable. F2B1 and F2B2 were positive-stable. These initial observations indicated that infection control and pig flow management techniques were sub-optimal, permitting PRRSV transmission among herds and age groups.

To begin eliminating PRRSV, LCH was initiated in F1B1 and F1B2. Herd closure avoided introducing PRRSV from external sites, and decreased the number of susceptible animals in the herds: both limiting viral transmission [17]. Simultaneous vaccination of all animals at both Week 0 and Week 4 increased herd immunity and may have promoted viral elimination by reducing the number of naïve animals. The vaccine used in this study is derived from a type 2 (North American) PRRSV strain, and its efficacy has been clearly demonstrated against both homologous and heterologous strains [28, 29].

The LCH model is a useful tool for PRRSV area elimination programs, and has repeatedly allowed control in individual farms [17, 22]. One of the limitations of LCH is the need for stringent biosecurity measures to prevent virus transmission. In this study, staff reviewed internal and external biosecurity procedures and implemented the 10GR, devised in 2005 by Boehringer Ingelheim, based on 10 years of field experience in controlling PRRSV spread. The 10GR are based on the principles of the McREBEL system for disease management [23], and were developed to simplify the McREBEL procedures and increase the likelihood of implementation. The 10GR are reported here for the first time.

The 10GR involved restricting the movement of pigs to prevent PRRSV transmission between age groups, and quarantining gilts before introducing them to breeding herds to avoid infecting them with PRRSV. F1WF1 was considered the most difficult farm from which to eliminate PRRSV because of its complex pig flow, which made implementing the 10GR difficult. Despite this difficulty, the 10GR were stringently followed in all herds, in both flows, at all times (except in F1WF2, which was unable to comply with rule 8), and this was ensured through regular auditing of all farms. This foundation of good management practice contributed to the success of PRRSV MLV vaccination and the LCH control model in eliminating PRRSV from the study area.

A study on transmission of PRRSV between herds in Ontario concluded that sharing herd ownership and transportation were among the most important factors for the spread of PRRSV between herds [30]. Indeed, sharing of personnel and transportation between F1B1, F1B2 and F1Q (under the same ownership) may have contributed to the endemicity of PRRSV in the Horne Peninsula before this study began. Although shared ownership may cause problems, it can also facilitate communication between producers, which is critical to the success of regional PRRSV control and elimination projects [31]. The naturally limited geographical area, the close relationship between the herd owners, and supervision of all herds by the same veterinarian probably contributed to the successful outcome of this study.

Using a combination of LCH, use of PRRSV type 2 MLV and the 10GR, PRRSV was successfully eliminated from F1B1 and F1B2 by January 2015. PCR positive pigs were detected in the nursery of F1B2 in November 2013, and most animals were exported away from the Horne Peninsula, or to quarantine in F1Q, but some presumed PRRSV negative pigs were moved to F1WF2. Unfortunately, these animals re-introduced PRRSV into F1WF2, and so having an emergency plan to remove infected pigs from the elimination area as soon as they are detected is a key learning from this study. We also suggest that extending the vaccination period of piglets at weaning to span a whole sow cycle (20 weeks) may have avoided the emergence of PRRSV positive pigs in F1B2. Genetic sequencing revealed that the virus strain had over 99% ORF-5 sequence homology to the PRRSV type 2 MLV strain. Although re-infection was disappointing, we were encouraged that field virus was not detected.

F1WF1 tested PRRSV ELISA and PCR negative in four sampling points over 6 months, but became re–infected in October 2014, at the same time that F1WF2 finisher rooms were depopulated following reinfection. F1WF1 and F1WF2 did not share personnel, transportation or equipment, so the infection in in F1WF1 may have been due to airborne transmission of PRRSV from F1WF2, less than 500 m away. Airborne transmission was previously shown under Danish field conditions [32], but no further investigations to confirm this were undertaken in the current study.

Depopulation of the oldest pigs in the nurseries of F2B1 and F2B2 helped to immediately disrupt transmission of PRRSV from nursery to finisher areas, as has been previously shown [33]. Despite depopulation, nurseries in breeding herds remained ELISA positive until September 2013, because piglets born to infected sows had maternal antibodies in serum. This was also the case in nurseries in F1B1 and F1B2, which also remained ELISA positive for several months after becoming PCR negative. A combination of depopulation and strict application of the 10GR led to the rapid elimination of PRRSV (ELISA and PCR negative) in F2B1 and F2B2 in just 2 months after study commencement, and declaration of PRRSV free SPF status 6 months later. Depopulation of the oldest pigs in nursery rooms of breeding herds enabled rapid PRRSV elimination from finisher herds too, by ensuring that no PRRSV positive piglets were introduced to finisher herds.

The authors note some limitations to the current study. To show that PRRSV area elimination is possible using the methods described, the Horne Peninsula was deliberately chosen as a limited geographical area, with few herd owners and simple transportation routes between herds. The breeding herds in this study were comparable in size and production to the Danish average in 2015 (742 sows and 22,077 piglets), while the finisher sites produced about half as many pigs as the Danish average for finisher sites (8008 pigs slaughtered in 2015) [34]. However, the authors acknowledge that elimination would be far more complex in less well defined areas. The current project was driven by a small number of stakeholders who dedicated time to planning and sampling. Extending this project to larger regions with more owners and increased animal transport would require substantially more planning. For example, empty barns would have to be identified so that PRRSV positive pigs could be moved from sites close to achieving PRRSV elimination, to prevent setbacks.

Furthermore, larger projects with more owners may encounter problems with commitment and communication. In this project, six veterinarians were involved with overseeing the study, and ensuring implementation of the 10GR. All but one of these veterinarians were from the same practice (Porcus Pig Practice), making the sharing of information and decisions simple. In larger projects, more stakeholders from different practices (and perhaps with competing interests) may make communication more difficult. Employment of a full-time project coordinator would be recommended, as would involvement of pig producers and representatives from SEGES Danish Pig Research Centre, slaughterhouses and SPF-Denmark.

PRRS is one of the most economically devastating swine diseases, causing substantial animal losses and medication expenses [35, 36]. In Denmark, the costs of PRRS are estimated to be between €4 and €139 per sow, per year [20]. The LCH method is an effective PRRSV elimination strategy when combined with stringent biosecurity measures: this was further confirmed in the present study. A detailed cost-benefit analysis is needed to understand the return on investment for this area PRRSV elimination method.

## Conclusions

PRRSV was eliminated from all herds on the Horne Peninsula, Denmark, in just over 18 months, after employing a combination of LCH, vaccination using PRRSV type 2 MLV and the 10GR for biosecurity management. Eighteen months later (November 2016), all herds still have PRRSV free SPF status. Elimination may have been achieved more quickly if the PRRSV positive pigs that were depopulated from F1B2 had been moved out of the area: this would have reduced the risk of area spread. Finally, the 10GR helped improve biosecurity management in all farms on the peninsula, and may offer a simplified alternative to the McREBEL system for controlling PRRSV.

## Additional files

**Additional file 1.** PCR and ELISA results from breeding herds 8 weeks before study commencement. Additional data showing individual PCR and ELISA results from piglets of different age groups in the breeding herds, 8 weeks before study commencement.

**Additional file 2.** Individual value plot of PRRS ELISA status of pre-wean piglets (3 weeks of age) in LCH breeding herds. Additional data showing ELISA S:P values on all sampling points for up to 90 weeks after implementation of LCH, measured in 3-week old piglets.

**Additional file 3.** PCR and ELISA results from F1WF1 (receiving piglets from LCH breeding herds) until 60 weeks after LCH commencement. Additional data showing individual PCR and ELISA results from piglets of different age groups on the WF1 site, throughout the entire study duration.

## Abbreviations

10GR: 10 Golden Rules
AIAO: all in, all out
ELISA: enzyme-linked immunosorbent assay
LCH: load, close, homogenise
McREBEL: Management Changes to Reduce Exposure to Bacteria to Eliminate Losses
MLV: modified–live vaccine
ORF: open reading frame
PCR: polymerase chain reaction
PRRS: porcine reproductive and respiratory syndrome
PRRSV: porcine reproductive and respiratory syndrome virus
SPF: specific pathogen free
