# Supplementary material for: Control and eradication of porcine reproductive and respiratory syndrome virus type 2 using a modified-live type 2 vaccine in combination with a load, close, homogenise model: an area elimination study
Source: Acta Vet Scand. 2017 Jan 5;59:4. doi: 10.1186/s13028-016-0270-z (PMC5217557; doi:10.1186/s13028-016-0270-z)
Supplement: Supplementary file 1 — Additional file 1. PCR and ELISA results from breeding herds 8 weeks before study commencement. Additional data showing individual PCR and ELISA results from piglets of different age groups in the breeding herds, 8 weeks before study commencement. [file 13028_2016_270_MOESM1_ESM.docx]

ADDITIONAL FILE 1

PCR and ELISA results from breeding herds 8 weeks before study commencement

|  |  | **F1B1** | | **F1B2** | | **F2B1** | | **F2B2** | |
| --- | --- | --- | --- | --- | --- | --- | --- | --- | --- |
|  |  | **ELISA^a^** | **PCR^b^** | **ELISA** | **PCR** | **ELISA** | **PCR** | **ELISA** | **PCR** |
| Age of piglets (weeks post-weaning | 0 | - | - | 100 | POS | 0 | NEG | 0 | NEG |
|  | 2 | - | - | 100 | POS | 0 | NEG | 0 | NEG |
|  | 3 | 100 | POS | 100 | POS | 0 | NEG | 0 | NEG |
|  | 4 | - | - | 100 | POS | 0 | NEG | 0 | NEG |
|  | 6 | - | - | 100 | POS | 0 | NEG | 0 | NEG |
|  | 7 | - | - | 100 | POS | 100 | POS | 100 | POS |
|  | 8 | - | - | 100 | POS | 100 | POS | 100 | POS |

a) ELISA results shown as percentage of samples that tested positive

b) PCR results shown as pooled serum samples either negative or positive for PRRSV

ELISA=enzyme-linked immunosorbent assay; NEG=negative; PCR=polymerase chain reaction; POS=positive
