# Supplementary material for: Control and eradication of porcine reproductive and respiratory syndrome virus type 2 using a modified-live type 2 vaccine in combination with a load, close, homogenise model: an area elimination study
Source: Acta Vet Scand. 2017 Jan 5;59:4. doi: 10.1186/s13028-016-0270-z (PMC5217557; doi:10.1186/s13028-016-0270-z)
Supplement: Supplementary file 2 — Additional file 2. Individual value plot of PRRS ELISA status of pre-wean piglets (3 weeks of age) in LCH breeding herds. Additional data showing ELISA S:P values on all sampling points for up to 90 weeks after implementation of LCH, measured in 3-week old piglets. [file 13028_2016_270_MOESM2_ESM.docx]

**ADDITIONAL FILE 2**

**Individual value plot of PRRS ELISA status of pre-wean piglets (3 weeks of age) in LCH breeding herds**


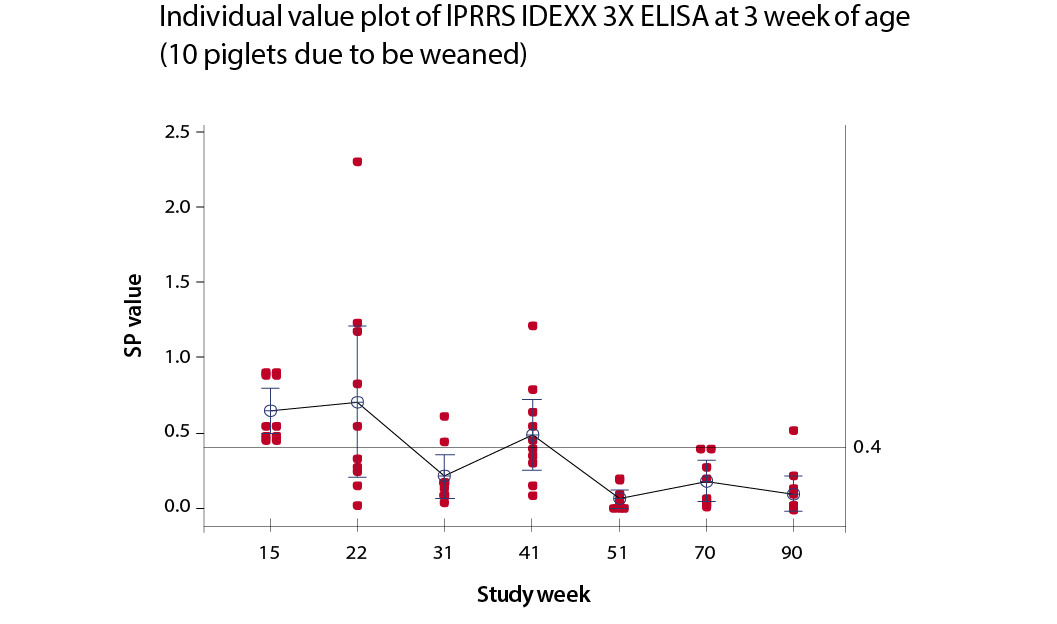


Data cut-off value was 1.4; Data are 95% confidence interval for the mean

ELISA=enzyme-linked immunosorbent assay; LCH=load, close, homogenise; PRRS=porcine reproductive and respiratory syndrome
