# Supplementary material for: Control and eradication of porcine reproductive and respiratory syndrome virus type 2 using a modified-live type 2 vaccine in combination with a load, close, homogenise model: an area elimination study
Source: Acta Vet Scand. 2017 Jan 5;59:4. doi: 10.1186/s13028-016-0270-z (PMC5217557; doi:10.1186/s13028-016-0270-z)
Supplement: Supplementary file 3 — Additional file 3. PCR and ELISA results from F1WF1 (receiving piglets from LCH breeding herds) until 60 weeks after LCH commencement. Additional data showing individual PCR and ELISA results from piglets of different age groups on the WF1 site, throughout the entire study duration. [file 13028_2016_270_MOESM3_ESM.docx]

ADDITIONAL FILE 3

PCR and ELISA results from F1WF1 (receiving piglets from the LCH breeding herds) until 60 weeks after LCH commencement

|  |  | **Jan-13** | | **Feb-14** | | **Mar-14** | | **May-14** | | **Jul-14** | | **Sep-14** | | **Oct-14** | | **Dec-14** | | February 2015 Depopulation | **Mar-15** | |
| --- | --- | --- | --- | --- | --- | --- | --- | --- | --- | --- | --- | --- | --- | --- | --- | --- | --- | --- | --- | --- |
|  |  | **ELISA^a^** | **PCR^b^** | **ELISA** | **PCR** | **ELISA** | **PCR** | **ELISA** | **PCR** | **ELISA** | **PCR** | **ELISA** | **PCR** | **ELISA** | **PCR** | **ELISA** | **PCR** |  | **ELISA** | **PCR** |
| Age of piglets (weeks) | 4 | 40 | NEG | - | - | - | - | 20 | NEG | 0 | NEG | - | - | 0 | NEG | 0 | NEG |  | 0 | NEG |
|  | 6 | 40 | NEG | - | - | 20 | NEG | 20 | NEG | - | - | - | - | - | - | - | - |  | - | - |
|  | 7 | - | - | - | - | - | - | - | - | 0 | NEG | 0 | NEG | 0 | NEG | 0 | NEG |  | 0 | NEG |
|  | 8 | 0 | NEG | 0 | NEG | 20 | NEG | 0 | NEG | - | - | - | - | - | - | - | - |  | - | - |
|  | 9 | - | - | - | - | - | - | - | - | 0 | NEG | 0 | NEG | 0 | NEG | 0 | NEG |  | - | - |
|  | 10 | 0 | NEG | 0 | NEG | 0 | NEG | 0 | NEG | - | - | - | - | - | - | - | - |  | - | - |
|  | 11 | - | - | - | - | - | - | - | - | 0 | NEG | 0 | NEG | 0 | NEG | 0 | POS |  | - | - |
|  | 12 | 0 | NEG | 0 | NEG | 0 | NEG | 20 | NEG | - | - | - | - | - | - | - | - |  | 0 | NEG |
|  | 13 | - | - | - | - | - | - | - | - | 0 | NEG | 0 | NEG | 0 | NEG | 100 | POS |  | - | - |
|  | 14 | 0 | NEG | - | - | 0 | NEG | 0 | NEG | - | - | - | - | - | - | - | - |  | - | - |
|  | 15 | - | - | - | - | - | - | - | - | 0 | NEG | 0 | NEG | 0 | NEG | 100 | POS |  | - | - |
|  | 16 | - | - | - | - | 100 | POS | - | - | - | - | - | - | - | - | - | - |  | 0 | NEG |
|  | 17 | - | - | 100 | NEG | - | - | - | - | - | - | 0 | NEG | 20 | POS | 0 | NEG |  | - | - |
|  | 18 | - | - | - | - | 50 | POS | - | - | - | - | - | - | 100 | POS | - | - |  | - | - |

a) ELISA results shown as percentage of samples that tested positive

b) PCR results shown as pooled serum samples either negative or positive for PRRSV

ELISA=enzyme-linked immunosorbent assay; NEG=negative; PCR=polymerase chain reaction; POS=positive
